# 3D U-Net for automated detection of multiple sclerosis lesions: utility of transfer learning from other pathologies

**DOI:** 10.3389/fnins.2023.1188336

**Published:** 2023-10-27

**Authors:** Stephen G. Wahlig, Pierre Nedelec, David A. Weiss, Jeffrey D. Rudie, Leo P. Sugrue, Andreas M. Rauschecker

**Affiliations:** ^1^Center for Intelligent Imaging (ci^2^), Department of Radiology and Biomedical Imaging, University of California, San Francisco, San Francisco, CA, United States; ^2^Department of Radiology, University of California, San Diego, San Diego, CA, United States

**Keywords:** multiple sclerosis, demyelination, deep learning, transfer learning, segmentation

## Abstract

**Background and purpose:**

Deep learning algorithms for segmentation of multiple sclerosis (MS) plaques generally require training on large datasets. This manuscript evaluates the effect of transfer learning from segmentation of another pathology to facilitate use of smaller MS-specific training datasets. That is, a model trained for detection of one type of pathology was re-trained to identify MS lesions and active demyelination.

**Materials and methods:**

In this retrospective study using MRI exams from 149 patients spanning 4/18/2014 to 7/8/2021, 3D convolutional neural networks were trained with a variable number of manually-segmented MS studies. Models were trained for FLAIR lesion segmentation at a single timepoint, new FLAIR lesion segmentation comparing two timepoints, and enhancing (actively demyelinating) lesion segmentation on T1 post-contrast imaging. Models were trained either de-novo or fine-tuned with transfer learning applied to a pre-existing model initially trained on non-MS data. Performance was evaluated with lesionwise sensitivity and positive predictive value (PPV).

**Results:**

For single timepoint FLAIR lesion segmentation with 10 training studies, a fine-tuned model demonstrated improved performance [lesionwise sensitivity 0.55 ± 0.02 (mean ± standard error), PPV 0.66 ± 0.02] compared to a de-novo model (sensitivity 0.49 ± 0.02, *p* = 0.001; PPV 0.32 ± 0.02, *p* < 0.001). For new lesion segmentation with 30 training studies and their prior comparisons, a fine-tuned model demonstrated similar sensitivity (0.49 ± 0.05) and significantly improved PPV (0.60 ± 0.05) compared to a de-novo model (sensitivity 0.51 ± 0.04, *p* = 0.437; PPV 0.43 ± 0.04, *p* = 0.002). For enhancement segmentation with 20 training studies, a fine-tuned model demonstrated significantly improved overall performance (sensitivity 0.74 ± 0.06, PPV 0.69 ± 0.05) compared to a de-novo model (sensitivity 0.44 ± 0.09, *p* = 0.001; PPV 0.37 ± 0.05, *p* = 0.001).

**Conclusion:**

By fine-tuning models trained for other disease pathologies with MS-specific data, competitive models identifying existing MS plaques, new MS plaques, and active demyelination can be built with substantially smaller datasets than would otherwise be required to train new models.

## 1. Introduction

Multiple sclerosis (MS) is a progressive neurodegenerative disease characterized by demyelinating lesions in the central nervous system and the leading cause of non-traumatic neurologic disability among young adults (Koch-Henriksen and Sørensen, 2010). It has an estimated global prevalence of 44 per 100,000 in 2020, increased from 29 per 100,000 in 2013 (Walton et al., 2020). Magnetic resonance imaging (MR) plays a key role in monitoring disease progression by identifying new lesions on T2-weighted or fluid-attenuated inversion recovery (FLAIR) sequences. Active demyelination is characterized by lesion enhancement on T1-weighted images following gadolinium-based contrast agent administration. Identification of new or actively demyelinating lesions can prompt clinicians to alter a patient’s treatment strategy.

MRIs of patients with MS often contain numerous lesions making the identification of new FLAIR lesions or tiny foci of enhancement a tedious and error-prone task that is ideally suited for computer-aided detection. Early MS segmentation algorithms used techniques such as region-growing algorithms (Heinonen et al., 1998; Wu et al., 2006), support vector machines (Lao et al., 2008; HosseiniPanah et al., 2019), random forest methods (Maier and Handels, 2015), and intensity-based outlier detection (Van Leemput et al., 2001). More recently the development of convolutional neural net (CNN) based algorithms has resulted in improved segmentation accuracy (Brosch et al., 2016; Aslani et al., 2019a,b; Coronado et al., 2020; Gabr et al., 2020; Krüger et al., 2020; McKinley et al., 2020, 2021; Narayana et al., 2020a,b; Fenneteau et al., 2021). In particular, networks based on the U-Net architecture with an encoder-decoder structure have yielded excellent results for segmentation of FLAIR lesions (Duong et al., 2019; La Rosa et al., 2020; Narayana et al., 2020a,b; Fenneteau et al., 2021; McKinley et al., 2021) and lesion enhancement (Coronado et al., 2020; Durso-Finley et al., 2020).

The identification of new or actively demyelinating MS plaques, rather than simply quantifying overall disease burden, is particularly important in guiding clinical management. As such, several groups have developed dedicated algorithms for segmentation of new FLAIR lesions on follow-up studies compared to an initial MR (Fartaria et al., 2019; Köhler et al., 2019; Schmidt et al., 2019; Krüger et al., 2020; McKinley et al., 2020; Salem et al., 2020). Some of these algorithms use a dedicated U-Net which incorporates both follow-up and baseline studies as inputs (Krüger et al., 2020; Salem et al., 2020), while others segment the follow-up and baseline studies individually and manipulate the resulting segmentation maps to generate a new lesion map (Köhler et al., 2019; Schmidt et al., 2019; McKinley et al., 2020).

One limitation of the above methods is that they used large amounts of manually segmented data to train their models, ranging from 50 to >1,000 MS studies (Coronado et al., 2020; Gabr et al., 2020; Narayana et al., 2020a; McKinley et al., 2021). Moreover, due to differences in scan parameters, technique, and patient populations, deep-learning models trained on one institution’s data are often not well suited to process another institution’s data (AlBadawy et al., 2018), limiting clinical utility. Prior work has demonstrated that re-training/fine-tuning these default models with even a small amount of institutional-specific data (i.e., transfer learning) can improve performance (Weeda et al., 2019; Rauschecker et al., 2022). However, it is currently unclear if transfer learning is effective when the default model is trained on non-MS imaging abnormalities from the same institution, given the different imaging appearance of small demyelinating plaques compared to other white matter pathologies such as leukodystrophy or gliomas.

In this work we evaluate the effect of transfer learning from models initially trained on other pathologies on MS segmentation efficacy. Since identification of new and actively demyelinating lesions are equally important goals in assessing lesion burden, we assess efficacy of new FLAIR lesion segmentation from paired initial and follow-up studies in addition to accuracy of single timepoint FLAIR and enhancement segmentation.

Each model is evaluated on its lesionwise sensitivity and positive predictive value (PPV), as these are the metrics most relevant to the interpreting radiologist. The value of sensitivity is easily apparent, as a high sensitivity model facilitates detection of patients with progressive disease. A high PPV is also necessary, as excessive false positives increase the radiologist’s interpretation time, which is a critical factor influencing utilization of deep learning tools in clinical practice. An increase in one of these metrics does not necessarily imply an increase in the other; in fact, a highly sensitive model may demonstrate a low PPV due to a large number of both true- and false-positive segmented lesions. As such, we also compare how sensitivity and PPV are differentially influenced by transfer learning.

## 2. Materials and methods

### 2.1. Patients and data

**Figure 1:**
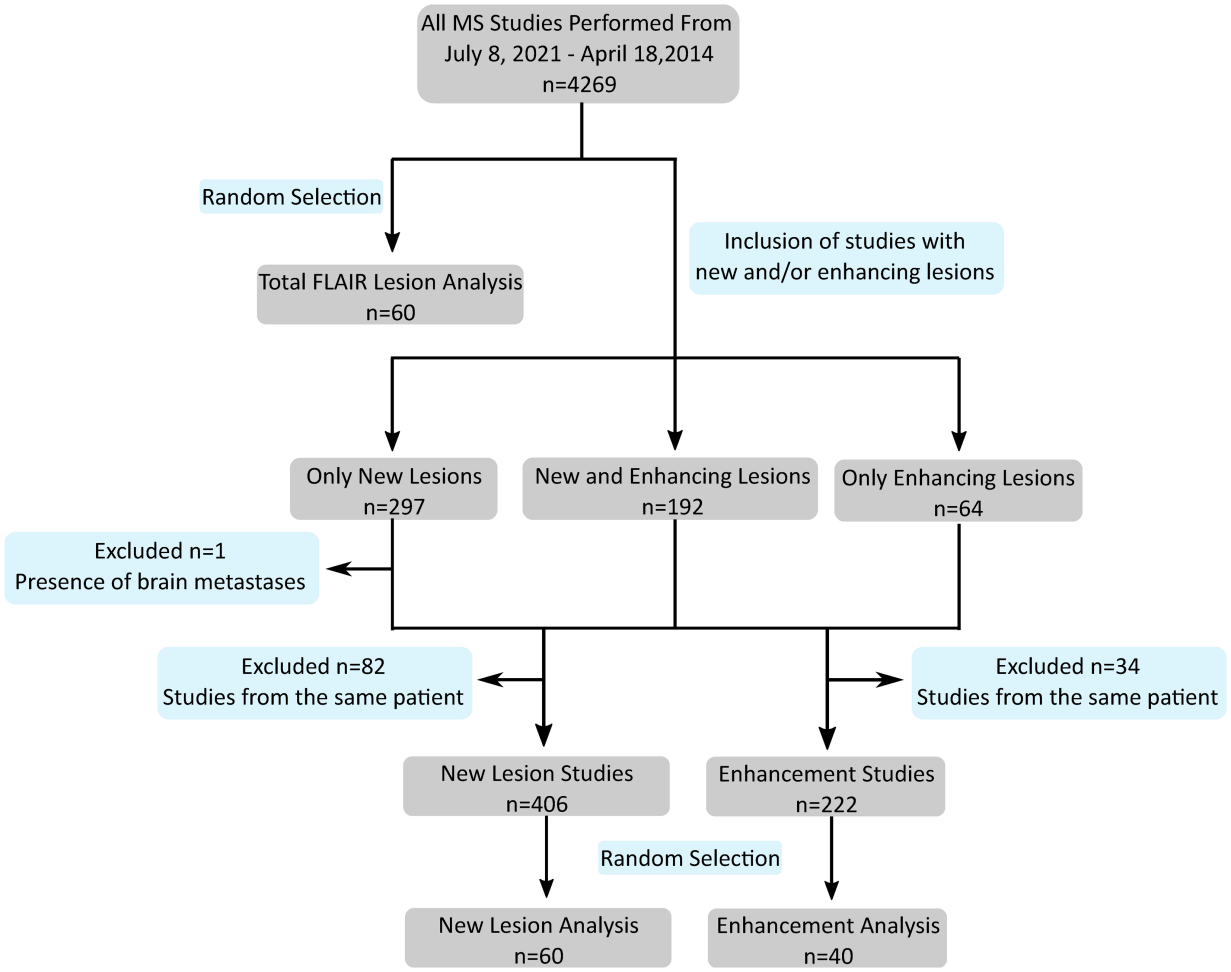
Flowchart shows study selection according to inclusion/exclusion criteria from initial search to the subset designated for analysis on each segmentation task (single timepoint FLAIR lesions, new lesions, and enhancement). *n*, number of studies; MS, multiple sclerosis; FLAIR, fluid attenuation inversion recovery.

This retrospective study was approved by the institutional review board of the University of California, San Francisco, with a waiver for consent due to minimal risk. Deidentified studies were obtained from our institution’s picture archiving and communication system (PACS) according to the procedure detailed in Figure 1. Inclusion criteria consisted of MRI studies ordered for follow-up or initial investigation of suspected multiple sclerosis, while exclusion criteria consisted of the presence of non-demyelinating enhancing intra-axial lesions or excessive motion artifact that impaired interpretation as specified in the diagnostic report. Additionally, for patients with multiple MS studies, only a single study or study pair was included in each analysis dataset. Of the 4,269 studies in the evaluated timeframe, 192 demonstrated both enhancing lesions and new lesions compared to a prior study while 297 demonstrated new lesions without enhancing lesions and 64 demonstrated enhancing lesions without new lesions (Figure 1). There was no minimum size of lesions. For single timepoint lesion analysis, one FLAIR sequence was used from each of 60 patients. For new lesion analysis, one FLAIR sequence was used from both an initial and follow-up timepoint from each of 60 patients. For enhancement segmentation, pre- and post-gadolinium 3D gradient recall echo (GRE) T1-weighted sequences were used from each of 40 patients. All studies were manually segmented by a radiologist referred to as Grader 1 (SGW, 3rd year radiology resident with 5+ years of image segmentation experience). A subset of 10 studies for single timepoint FLAIR lesion analysis were also jointly segmented by two additional radiologists (JDR and AMR, attending neuroradiologists with 2 and 3 years of post-residency experience, respectively) for comparison purposes; each individual study was segmented by only one of JDR or AMR. This segmentation set is referred to as Grader 2. Grader 1 was used as the ground truth for statistical analysis, including evaluation of Grader 2.

### 2.2. Default models

The default single timepoint FLAIR U-Net (Duong et al., 2019) was trained on 34 MR studies from the University of California, San Francisco and 293 MR studies from the University of Pennsylvania, including various underlying pathologies previously described, all demonstrating lesions of varying shape and size with hyperintense FLAIR signal abnormality. Pathologies ranged from tumors to leukoencephalopathy to chronic small vessel ischemia. The default FLAIR U-Net to detect new lesions was trained on 198 patients with brain tumors with consecutive imaging and manual segmentations delineating areas of change on FLAIR imaging (Rudie et al., 2022). The default enhancement U-Net was trained on 463 MR studies demonstrating abnormally enhancing metastatic tumors (“metastases”) from the University of California, San Francisco (Rudie et al., 2021). All studies were obtained with approval from the institutional review board from the respective institute.

### 2.3. Deep learning segmentation

The same 3D U-Net architecture was used for all models unless otherwise specified, both de-novo and fine-tuned. All images were pre-processed as described elsewhere (Duong et al., 2019; Rauschecker et al., 2020; Rudie et al., 2021). In brief, images were normalized by the mean and standard deviation (SD) signal intensity to zero mean and unit SDs and resampled to 1 mm^3^ isotropic resolution *via* linear interpolation. A variety of elastic transformations (Simard et al., 2003) were applied for data augmentation. Each augmented imaging volume was subsequently split into 96-mm^3^ 3D patches for input to the network. During training, the patches were randomly sampled across the full-brain volume. A total of 60 patches, split into 30 lesion-inclusive and 30 lesion-exclusive patches, were obtained from each training MRI and subject to 3 augmentations, resulting in 180 patches per MRI.

**Figure 2:**
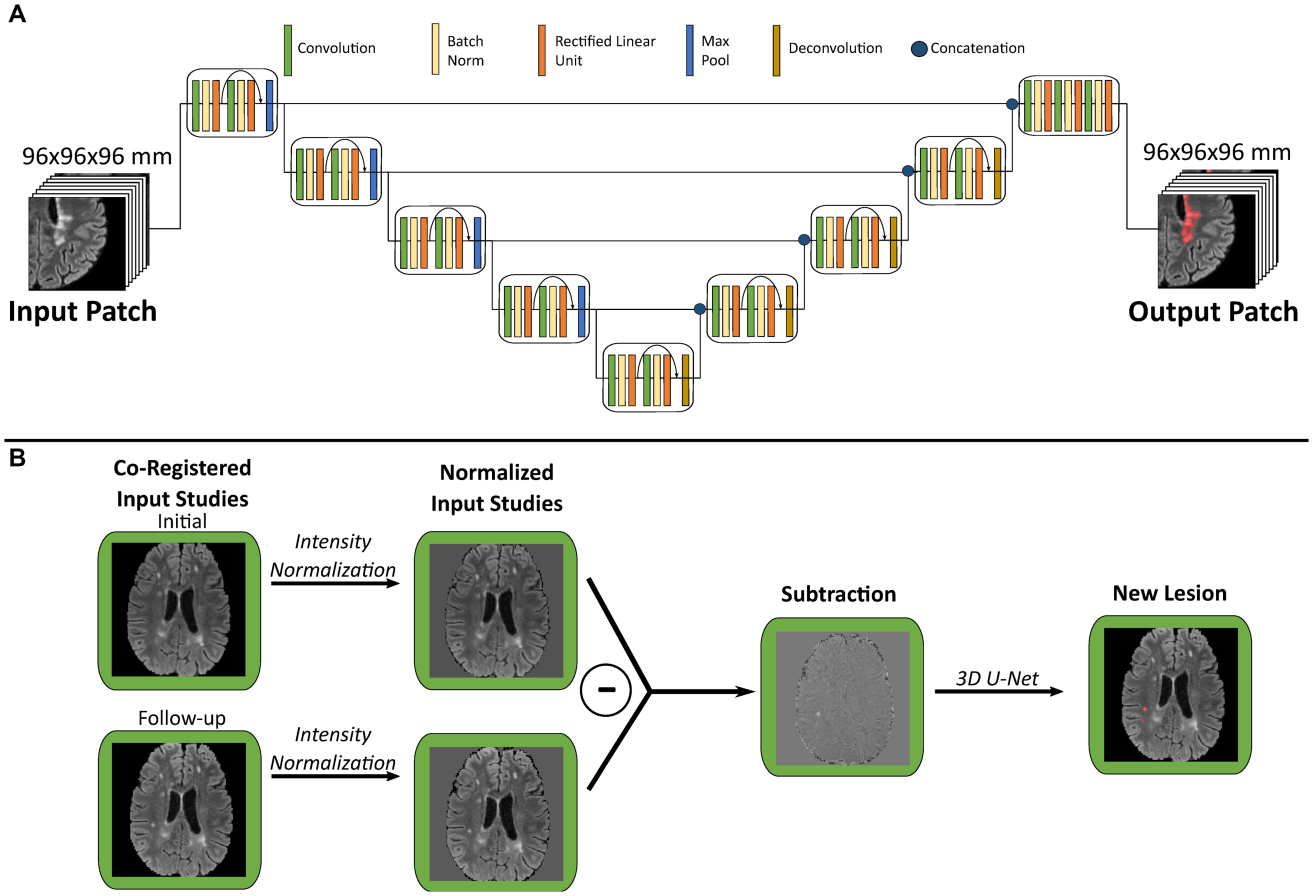
Graphical overview of multiple sclerosis analysis algorithm. **(A)** Schematic of three-dimensional U-Net architecture used for both FLAIR and enhancement segmentation. **(B)** Illustration of new lesion analysis of FLAIR images for a single patient. Each pair of co-registered initial and follow-up studies are used to generate a subtraction volume, which is then processed through a 3D U-Net to segment new lesions. FLAIR, fluid attenuation inversion recovery.

Following image pre-processing, both FLAIR and enhancing lesions were detected with our previously developed CNN networks with three-dimensional U-Net architecture (3D U-Net, Figure 2A) using FLAIR and T1 pre/post-gadolinium images as inputs, respectively. The U-Net consists of 4 down-sampled blocks followed by 4 up-sampled blocks. Training was performed for 30 epochs with a standard cross-entropy loss, Adam optimizer, and learning rate of 10^−5^; further details on architecture and training process are described in Duong et al. (2019). To develop MS-specific models, we used MS training data to fine-tune our default disease-invariant FLAIR model (Duong et al., 2019), glioma-specific new FLAIR signal model (Rudie et al., 2022), and metastases-specific enhancement model (Rudie et al., 2021). We compared these fine-tuned models with de-novo models trained with the same data.

As an additional comparison, the single timepoint FLAIR segmentation experiment was repeated with two alternate U-Net architectures. One alternate architecture was shallower than the original and contained only 3 down-sampled blocks (Supplementary Figure S1A), while the other alternate architecture was deeper and contained 5 down-sampled blocks (Supplementary Figure S2A). All other components of these alternate architectures were unchanged from the original described above.

### 2.4. New lesion analysis

Our algorithm for new lesion segmentation relies upon generation of a single subtraction volume (Figure 2B) generated from the input of an initial/follow-up study pair. First, the follow-up FLAIR study is registered to the initial study using a symmetric normalization transformation with Advanced Normalization Tools (ANTs;^1^ RRID:SCR_004757), followed by signal intensity normalization and re-sampling as described above. Subsequently the initial study image is subtracted from the follow-up study image to create a subtraction volume, which highlights the intensity differences between each study pair. This subtraction volume is then used as input to our 3D U-Net for both training and prediction of new lesions. Of note, our glioma-specific default model was trained using subtraction volume inputs, while the original model described in Rudie et al. used a multi-channel architecture with both timepoint FLAIR volumes as separate inputs (Rudie et al., 2022).

### 2.5. Outside institution test set

Our single timepoint FLAIR lesion models were used to segment lesions in MRI studies from the publicly-available ISBI 2015 dataset (Carass et al., 2017). Since human reference segmentations were only available for the training dataset (21 studies from 5 patients), the official test set was excluded from analysis. Segmentation performance was evaluated both without training on any ISBI data and after fine-tuning on a subset of ISBI studies. A leave-one-out cross validation was performed in which the models were trained on four patients’ studies and tested on the remaining patient.

### 2.6. Statistical analysis

For each segmentation task, the total dataset was split into 4 (for enhancement segmentation) or 6 (for single timepoint and new lesion FLAIR segmentation) subsets of 10 studies each. The first subset of 10 studies was used to test the default model, which facilitated comparison with the independent human segmentation (Grader 2). All other models were assessed on the entire dataset *via* a 4 or 6-fold cross-validation approach. For example, when evaluating models that use 10 training studies, one fine-tuned model was trained on the first subset of 10 studies and tested on the second subset of 10 studies, while another fine-tuned model was trained on the second subset and tested on the third subset. This pattern was repeated until all subsets had been used as the test set. This algorithm is depicted in graphical form in Supplementary Figure S3.

All analyses were performed in study native space. Lesion detection performance was compared using lesionwise sensitivity [mean ± standard error (SE)] and positive predictive value (PPV; mean ± SE). Data was averaged across all folds of the cross validation. Evaluation of performance across methods was accomplished with one-way ANOVA with repeated measures followed by paired 2-tailed t-tests to compare the de-novo and fine-tuned training approaches. Unpaired 2-tailed t-tests were used for comparison with the default models and Grader 2 segmentation. All statistical operations were corrected for multiple comparisons with the Bonferroni correction.

## 3. Results

### 3.1. Patient demographics

A total of 149 patients (110 women, age range, 15–70 years, median age, 40 years) were included (Tables 1, 2). For single timepoint FLAIR lesion analysis the training set had a range of 2–216 lesions per patient (median 26) compared to 15–58 (median 34.5) for the test set. For new lesion analysis the training set had a range of 1–64 new lesions per patient (median 3) compared to 1–14 (median 3) for the test set. For post-contrast enhancement analysis the training set had a range of 1–18 enhancing lesions per patient (median 2) compared to 1–6 (median 3) for the test set.

**Table 1:** Patient demographics.

			FLAIR single timepoint training	FLAIR single timepoint testing	FLAIR serial timepoint training	FLAIR serial timepoint testing	Enhancement training	Enhancement testing
Patient number			50	10	50	10	30	10
Sex	Female		35 (70%)	8 (80%)	37 (74%)	8 (80%)	23 (77%)	8 (80%)
	Male		15 (30%)	2 (20%)	13 (26%)	2 (20%)	7 (23%)	2 (20%)
Age (years)		Initial	45 ± 12	49 ± 12	37 ± 11	42 ± 12	35 ± 11	39 ± 12
		Follow-up			38 ± 11	44 ± 13		
Field strength*	3 T	Initial	44 (88%)	7 (70%)	35 (70%)	6 (60%)	26 (87%)	10 (100%)
		Follow-up			37 (74%)	7 (70%)		
	1.5 T	Initial	6 (12%)	3 (30%)	15 (30%)	4 (40%)	4 (13%)	0
		Follow-up			13 (26%)	3 (30%)		
TE (msec)^#^	Maximum	Initial	388	495	394	388	16	3
		Follow-up			455	143		
	Median	Initial	130	148	127	131	3	3
		Follow-up			116	116		
	Minimum	Initial	99	95	127	116	2	2
		Follow-up			116	98		
TR (msec)^#^	Maximum	Initial	7,202	10,000	10,000	10,000	2,451	2,450
		Follow-up			7,202	6,952		
	Median	Initial	6,000	5,650	5,952	5,959	626	2,300
		Follow-up			6,002	6,252		
	Minimum	Initial	5,000	4,002	4,600	5,000	4	5
		Follow-up			4,000	5,800		
FLAIR acquisition	3D	Initial	46 (92%)	10 (100%)	42 (84%)	9 (90%)		
		Follow-up			50 (100%)	10 (100%)		
	2D	Initial	4 (8%)	0	8 (16%)	1 (10%)		
		Follow-up			0	0		

Unless otherwise specified, data are the number of patients in each category, with percentages in parentheses. Age is listed as mean ± standard deviation. TE and TR values are listed for FLAIR sequences and post-gadolinium 3D GRE T1-weighted sequences. FLAIR, fluid attenuation inversion recovery; TE, echo time; TR, repetition time; GRE, gradient recall echo.

**Table 2:** Scanner models.

Scanner manufacturer	Model	FLAIR single timepoint training	FLAIR single timepoint testing	FLAIR serial timepoint training (initial/follow-up)	FLAIR serial timepoint testing (initial/follow-up)	Enhancement training	Enhancement testing
GE	Signa Premier	8 (16%)	1 (10%)	4 (8%)/12 (24%)	1 (10%)/2 (20%)	7 (23%)	3 (30%)
	Signa HDxt	6 (12%)	3 (30%)	13 (26%)/13 (26%)	2 (20%)/3 (30%)	3 (10%)	0
	Signa Architect	0	0	0/1 (2%)	0/0	0	0
	Signa Artist	0	0	1 (2%)/0	0/0	0	0
	Signa PET/MR	0	0	1 (2%)/0	1 (10%)/3 (30%)	0	1 (10%)
	Discovery MR750	3 (6%)	0	14 (28%)/12 (24%)	1 (10%)/0	4 (13%)	2 (20%)
	Discovery MR750w	6 (12%)	3 (30%)	1 (2%)/6 (12%)	2 (20%)/2 (20%)	3 (10%)	0
	Optima MR450w	0	0	1 (2%)/0	0/0	0	0
Siemens	Skyra	25 (50%)	3 (30%)	11 (22%)/6 (12%)	1 (10%)/0	12 (40%)	4 (40%)
	Verio	1 (2%)	0	0/0	1 (10%)/0	1 (3%)	0
Philips	Intera	0	0	0/0	1 (10%)/0	0	0
	Ingenia	1 (2%)	0	2 (4%)/0	0/0	0	0
	Achieva	0	0	2 (4%)/0	0/0	0	0

Data are the number of studies in each category, with percentages in parentheses. FLAIR, fluid attenuation inversion recovery.

### 3.2. Single timepoint FLAIR lesion analysis

**Figure 3:**
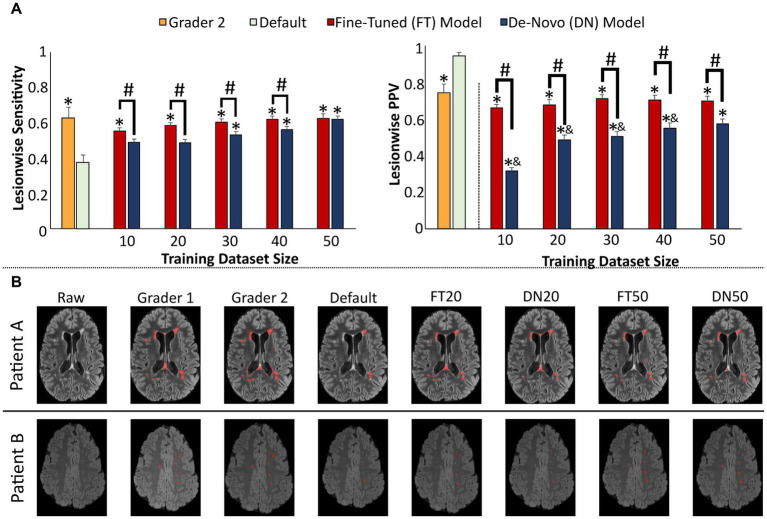
Performance of single timepoint FLAIR lesion prediction models. **(A)** Model performance assessed by lesionwise sensitivity and PPV statistics across the test set, compared to a second human observer (Grader 2, orange) and compared to the default disease-invariant model (green). **(B)** Images showing representative FLAIR slices with predicted new lesion maps. Error bars in each bar graph represent ± 1 standard error of the mean across patients. *p < 0.05 in comparison with the default FLAIR model after multiple comparison correction. &*p* < 0.05 in comparison with the Grader 2 segmentation after multiple comparison correction. #*p* < 0.05 in comparison with alternative training paradigm (fine-tune vs. de-novo model) using the same training dataset size, after multiple comparison correction. FLAIR, fluid attenuation inversion recovery; PPV, positive predictive value.

The first model was trained to generate segmentations of all MS lesions at a single timepoint on FLAIR imaging (Table 3). The default model trained on non-MS pathology suffered from low lesion sensitivity (0.38 ± 0.04 [mean ± SE]) compared to independent human segmentations (Grader 2, sensitivity 0.63 ± 0.06, *p* = 0.003). However, as few as 20 MS studies used to fine-tune this default model allowed improvement in sensitivity (0.59 ± 0.02, *p* = 0.515 [compared to Grader 2]) and PPV (0.68 ± 0.03, *p* = 0.272) to human performance levels, with only marginal gain from additional training studies (Figure 3A). With 50 training studies both the fine-tuned (0.63 ± 0.03) and de-novo models (0.62 ± 0.02) demonstrated similar sensitivity compared to independent human segmentation (Grader 2; 0.63 ± 0.06), although this de-novo model still yields a lower PPV (0.57 ± 0.03) than either the fine-tuned (0.70 ± 0.03, *p* < 0.001) or independent human (0.74 ± 0.05, *p* = 0.008) segmentations.

**Table 3:** Single timepoint FLAIR segmentation model performance.

Model	Training method	Lesionwise sensitivity	*p* ^Default^	*p* ^Grader2^	*p* ^Method^	Lesionwise PPV	*p* ^Default^	*p* ^Grader2^	*p* ^Method^
Default		0.38 ± 0.04				0.95 ± 0.02			
Grader 2		0.63 ± 0.06	0.003*			0.74 ± 0.05	0.001*		
10	FT	0.55 ± 0.02	0.001*	0.260	0.001*	0.66 ± 0.02	<0.001*	0.143	<0.001*
	DN	0.49 ± 0.02	0.023	0.044		0.32 ± 0.02	<0.001*	<0.001*	
20	FT	0.59 ± 0.02	<0.001*	0.515	<0.001*	0.68 ± 0.03	<0.001*	0.272	<0.001*
	DN	0.49 ± 0.02	0.031	0.040		0.49 ± 0.03	<0.001*	<0.001*	
30	FT	0.60 ± 0.02	<0.001*	0.700	0.002*	0.71 ± 0.02	<0.001*	0.569	<0.001*
	DN	0.53 ± 0.02	0.003*	0.153		0.50 ± 0.03	<0.001*	0.001*	
40	FT	0.62 ± 0.02	<0.001*	0.892	0.003*	0.70 ± 0.02	<0.001*	0.499	0.001*
	DN	0.56 ± 0.02	0.001*	0.311		0.55 ± 0.03	<0.001*	0.004*	
50	FT	0.63 ± 0.03	<0.001*	0.978	0.614	0.70 ± 0.03	<0.001*	0.434	<0.001*
	DN	0.62 ± 0.02	<0.001*	0.884		0.57 ± 0.03	<0.001*	0.008	

All sensitivity and PPV values are displayed as mean ± standard error. FT, fine-tuned; DN, de-novo; PPV, positive predictive value; *p*^Default^, *p* value from comparison with the default model; *p*^Grader2^, *p* value from comparison with the Grader 2 segmentation; *p*^Method^, *p* value from comparison between fine-tuned and de-novo training methods with the same training dataset size. *Indicates statistical significance (*p* < 0.05 after accounting for Bonferroni correction).

Representative slices from test studies with accompanying segmentations (Figure 3B) demonstrate that nearly all models perform well on the classic ovoid periventricular demyelinating lesions without a marked difference between the fine-tuned and de-novo models. However, the MS-trained models more consistently segment smaller juxtacortical lesions than the default model; performance on these lesions also improves with increasing training dataset size.

Performance on the external 2015 ISBI dataset demonstrated comparable results to previously published segmentation algorithms (Table 4). The fine-tuned models are again seen to outperform de-novo models when using small training datasets, particularly with respect to lesionwise PPV. Additional fine-tuning with a portion (4 patients) of the ISBI dataset further increased performance on the remainder of the ISBI dataset, as would be expected from prior results (Rauschecker et al., 2022; Table 4). However it should be acknowledged that even fine-tuned model performance falls short of the best algorithms tested on the ISBI dataset (Aslani et al., 2019a); this may reflect limitations related to use of a default model trained on non-MS data, small training datasets, and/or differences in neural net architecture.

**Table 4:** Model performance on International Symposium on Biomedical Imaging (ISBI) 2015 dataset.

Model	Dice score	Lesionwise sensitivity	Lesionwise PPV
**No ISBI-specific training**			
De-novo 10	0.53	0.65	0.13
Fine-tuned 10	0.60	0.61	0.29
De-novo 30	0.62	0.57	0.39
Fine-tuned 30	0.63	0.53	0.66
De-novo 50	0.63	0.59	0.46
Fine-tuned 50	0.62	0.56	0.61
**Cross validation with ISBI data (train 4/test 1)**			
De-novo 10	0.65	0.62	0.34
Fine-tuned 10	0.66	0.57	0.75
De-novo 30	0.66	0.58	0.57
Fine-tuned 30	0.66	0.56	0.72
De-novo 50	0.66	0.53	0.64
Fine-tuned 50	0.65	0.54	0.75
**Comparison models**			
(Maier and Handels, 2015)	0.70	0.53	0.52
(Brosch et al., 2016)	0.68	0.75	0.45
(Aslani et al., 2019b)	0.70	0.75	0.52
(Aslani et al., 2019a)	0.76	0.67	0.88

Models under “No ISBI-Specific Training” were evaluated directly on the test data. The cross-validation model was further fine-tuned with four patients’ studies from the ISBI dataset prior to evaluation. PPV, positive predictive value.

This single timepoint FLAIR segmentation experiment yielded similar results when repeated using alternate shallower (Supplementary Figure S1B; Supplementary Table S1) and deeper U-Net architectures (Supplementary Figure S2B; Supplementary Table S2), with fine-tuned models demonstrating significantly greater PPV than the de-novo model comparisons across all training dataset sizes. These results suggest that the value of transfer learning is not specific to a single neural network architecture.

### 3.3. New FLAIR lesion analysis

**Figure 4:**
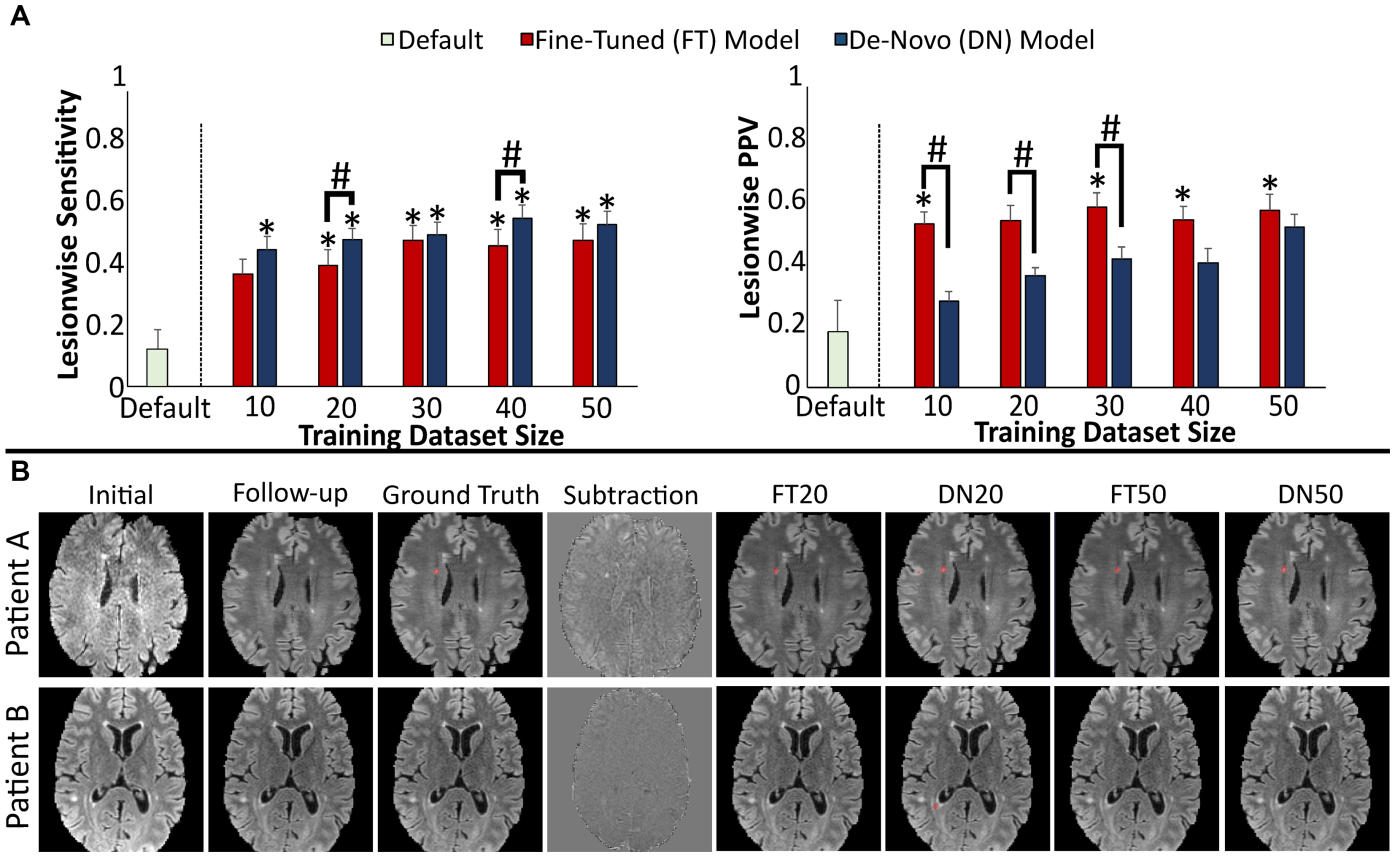
Performance of models detecting new lesions on FLAIR images across two timepoints. **(A)** Model performance for detection of new lesions assessed by lesionwise sensitivity and PPV. **(B)** Images showing representative FLAIR slices with predicted new lesion maps. Error bars in each bar graph represents ± 1 standard error of the mean across cases. *p < 0.05 in comparison with the default FLAIR model after multiple comparison correction. #*p* < 0.05 in comparison with alternative training paradigm (fine-tune vs. de-novo model) using the same training dataset size, after multiple comparison correction. FLAIR, fluid attenuation inversion recovery, PPV, positive predictive value.

The second model was trained to identify new demyelinating lesions from FLAIR MRI at one timepoint compared to a prior timepoint (Table 5). The default new FLAIR lesion model was originally trained on serial post-treatment gliomas studies for the purpose of identifying new geographic regions of signal abnormality (Rudie et al., 2022). While this is a very different task than identifying discrete new demyelinating lesions, we were interested in evaluating the merit of transfer learning from one pathology to another on this subtraction-based longitudinal assessment model. This default model suffered from poor lesionwise sensitivity (0.13 ± 0.07) and PPV (0.18 ± 0.11). However, PPV in particular benefited from fine-tuning on MS-specific data, even with only 10 training studies (fine-tuned 0.54 ± 0.04, *p* = 0.005 [compared to default model]; de-novo 0.29 ± 0.03, *p* = 0.360) but further increasing with more training data (Figure 4A). The de-novo trained model had higher sensitivity for lesions overall than the fine-tuned model (although this difference was only statistically significant with 20 (fine-tuned 0.41 ± 0.05, de-novo 0.50 ± 0.04, *p* = 0.003) and 40 training studies (fine-tuned 0.48 ± 0.06, de-novo 0.57 ± 0.04, *p* = 0.010)), and both the fine-tuned and de-novo models demonstrated higher sensitivity than the default model.

**Table 5:** New FLAIR lesion segmentation model performance.

Model	Training method	Lesionwise sensitivity	*p* ^Default^	*p* ^Method^	Lesionwise PPV	*p* ^Default^	*p* ^Method^
Default		0.13 ± 0.07			0.18 ± 0.11		
10	FT	0.38 ± 0.05	0.007	0.033	0.54 ± 0.04	0.005*	0.002*
	DN	0.46 ± 0.04	0.001*		0.29 ± 0.03	0.360	
20	FT	0.41 ± 0.05	0.003*	0.003*	0.55 ± 0.05	0.006	0.002*
	DN	0.50 ± 0.04	<0.001*		0.37 ± 0.03	0.103	
30	FT	0.49 ± 0.05	<0.001*	0.437	0.60 ± 0.05	0.002*	0.002*
	DN	0.51 ± 0.04	<0.001*		0.43 ± 0.04	0.046	
40	FT	0.48 ± 0.06	0.001*	0.010*	0.56 ± 0.05	0.005*	0.014
	DN	0.57 ± 0.04	<0.001*		0.42 ± 0.05	0.063	
50	FT	0.49 ± 0.06	0.001*	0.161	0.59 ± 0.05	0.003*	0.157
	DN	0.55 ± 0.05	<0.001*		0.53 ± 0.04	0.007	

All sensitivity and PPV values are displayed as mean ± standard error. FT, fine-tuned; DN, de-novo; PPV, positive predictive value; *p*^Default^, *p* value from comparison with the default model; *p*^Method^, *p* value from comparison between fine-tuned and de-novo training methods with the same training dataset size. *Indicates statistical significance (*p* < 0.05 after accounting for Bonferroni correction).

Representative slices from test studies with accompanying segmentations (Figure 4B) further illustrates that the effect of transfer learning and increasing training dataset size is reflected in fewer false positive segmentations. In particular, the de-novo models were more likely to include false positive segmentations along the boundaries of unchanged lesions and cortex. Conversely, the fine-tuned models were more likely to miss lesions in these areas; we suspect this is related to increased registration-related noise associated with sharp boundaries between low and high FLAIR signal (Table 6).

**Table 6:** Enhancing lesion segmentation model performance.

Model	Training method	Lesionwise sensitivity	*p* ^Default^	*p* ^Method^	Lesionwise PPV	*p* ^Default^	*p* ^Method^
Default		0.73 ± 0.09			0.39 ± 0.08		
10	FT	0.68 ± 0.06	0.649	0.001*	0.67 ± 0.04	0.003*	<0.001*
	DN	0.39 ± 0.05	0.004*		0.27 ± 0.04	0.122	
20	FT	0.74 ± 0.06	0.948	0.001*	0.69 ± 0.05	0.003*	0.001*
	DN	0.44 ± 0.09	0.028		0.37 ± 0.05	0.767	
30	FT	0.72 ± 0.05	0.881	0.001*	0.58 ± 0.03	0.018	0.078
	DN	0.46 ± 0.06	0.020		0.45 ± 0.07	0.564	

All sensitivity and PPV values are displayed as mean ± standard error. FT, fine-tuned; DN, de-novo; PPV, positive predictive value; *p*^Default^, *p* value from comparison with the default model; *p*^Method^, *p* value from comparison between fine-tuned and de-novo training methods with the same training dataset size. *Indicates statistical significance (*p* < 0.05 after accounting for Bonferroni correction).

### 3.4. Enhancing (actively demyelinating) lesion analysis

**Figure 5:**
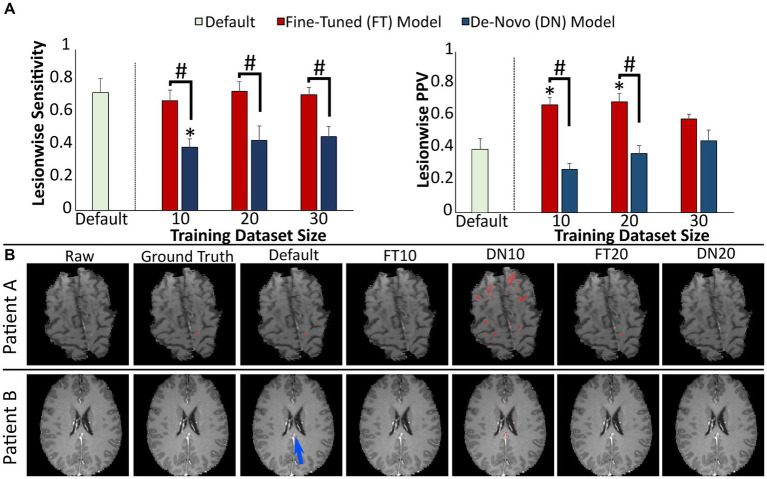
Performance of prediction models for MS lesion enhancement. **(A)** Model performance for detection of enhancing lesions assessed by lesionwise sensitivity and PPV. **(B)** Images showing representative slices from T1 post-gadolinium images with segmentations from each prediction model. Blue arrow highlights a small false positive segmentation along the falx. Error bars in each bar graph represent ± 1 standard error of the mean across cases. *p < 0.05 in comparison with the default enhancement model after multiple comparison correction. #*p* < 0.05 in comparison with alternative training paradigm (fine-tuned vs. de-novo model) using the same training dataset size, after multiple comparison correction. MS, multiple sclerosis; PPV, positive predictive value.

Gadolinium contrast agents are used in most studies of MS in order to highlight lesions with active demyelination. The third model was trained to identify these enhancing lesions on T1 post-contrast imaging. The default enhancement model was trained to detect enhancing brain lesions in the context of intracranial metastases (Rudie et al., 2021). This task is computationally similar to enhancing MS lesion detection because both of these pathologies demonstrate abnormal enhancement and are typically focal lesions of a similarly small size. This default model demonstrated high sensitivity (0.73 ± 0.09) but relatively low PPV (0.39 ± 0.08) Table 6. The PPV was again greatly improved when fine-tuning the model on even a small number of MS-specific cases, as few as 10 studies (fine-tuned 0.67 ± 0.04, *p* = 0.003 [compared to default model]; de-novo 0.27 ± 0.04, *p* = 0.122), without a significant decrease in lesionwise sensitivity [fine-tuned 0.68 ± 0.06, *p* = 0.649 (compared to default model); de-novo 0.39 ± 0.05, *p* = 0.004; Figure 5A].

Representative slices from test studies with accompanying segmentations (Figure 5B) illustrate the fine-tuned models’ higher performance, particularly with regard to fewer false positive segmentations. For example, the de-novo models were more likely to include false positive segmentations adjacent to normally enhancing structures such as the falx or dural venous sinuses.

## 4. Discussion

Despite the proven efficacy of deep learning algorithms in segmenting MS lesions, these techniques have yet to become widely used in clinical practice. There are multiple reasons for this delay in clinical implementation, but one challenge is that many of these published algorithms require a large amount of manually segmented training data (Coronado et al., 2020; Gabr et al., 2020; Narayana et al., 2020a), only to suffer in performance when applied to a new institution’s dataset. Transfer learning is a well-described technique used to mitigate the need for such large training datasets when a pre-trained default model is available (Weeda et al., 2019). However, it is unclear whether this benefit still applies when the default model is initially trained on non-MS pathology. Additionally, in clinical practice the detection of new or actively demyelinating lesions is often more important than cataloging each individual FLAIR lesion. As such, we describe the application of transfer learning to multiple non-MS default models for segmentation of existing MS lesions on single timepoint FLAIR imaging, new MS plaques on serial FLAIR imaging, and actively demyelinating lesions on T1 post-contrast imaging.

For segmentation of FLAIR lesions at a single timepoint, our results show that a model initially trained to segment a variety of FLAIR hyperintense pathologies and then fine-tuned with 10–30 MS studies demonstrates performance superior to a de-novo model and comparable to a human observer. While there was a potential benefit in segmentation performance with larger training datasets, this study did not include sufficient data to draw firm conclusions regarding optimal training dataset sizes. Instead, the primary take-away is that if only small amounts of institution-specific manually-labeled MS data are available, then the transfer learning approach is superior to building a de-novo model, saving valuable time and resources in manually labeling data.

For segmentation of new FLAIR lesions across two timepoints, the models trained on longitudinal glioma analysis and fine-tuned on MS lesions demonstrated higher PPV across all training dataset sizes while the de-novo models demonstrated higher sensitivity. This difference likely reflects the underlying characteristics of the default model for detecting new lesions, which was trained to detect changes in geographic areas of FLAIR signal abnormality associated with gliomas. New demyelinating lesions are far smaller than regions of glioma-associated FLAIR abnormality and as such it is understandable that a model trained exclusively on MS-lesions would demonstrate superior sensitivity. Yet even though lesionwise sensitivity is a critical performance metric for clinical applications, the significant improvement in PPV demonstrated by the fine-tuned models suggests that transfer learning may still have value despite the absence of an ideal default model.

The favorable impact of transfer learning was most apparent in segmentation of enhancing lesions; with 10 training studies the model trained on metastatic tumors and fine-tuned on enhancing MS lesions demonstrated a > 70% relative increase in sensitivity and PPV compared to the corresponding de-novo model. We suspect these substantial gains are related to the statistical image similarities between enhancing MS lesions and the enhancing metastases used to train this default model, despite their profoundly differing underlying pathologies. More specifically, brain MRIs demonstrating metastases often contain numerous small enhancing lesions, which exposes the deep learning model to a high number of training lesions. Since enhancing multiple sclerosis lesions are similar to enhancing metastases in size and appearance, this robust initial training yields a very sensitive default model. In contrast, however, most multiple sclerosis studies with active disease contain 1–3 enhancing lesions. The use of this MS-specific training data results in a significantly increased PPV compared to the default model. Conversely, the single timepoint and new lesion default models were trained on pathologies with few large areas of signal abnormality such as gliomas, leukodystrophy, or lymphoma and correspondingly demonstrated higher PPV than the de-novo models with small training datasets. For these single timepoint and new lesion tasks, use of MS-specific data with numerous small lesions results in significantly increased sensitivity compared to the default models.

Our study has limitations. For both FLAIR and enhancing lesion segmentation, it is likely that use of larger training datasets would further improve performance. Our goal was to demonstrate the relative impact of transfer learning on segmentation performance, which is most apparent with relatively few training studies. However, due to this limitation our models would likely underperform in direct comparison to models with more training data (Coronado et al., 2020; Gabr et al., 2020; Narayana et al., 2020a,b) or a more advanced underlying network architecture (Krüger et al., 2020; McKinley et al., 2020; Salem et al., 2020). Additionally, our glioma-specific default model used for new lesion detection is poorly suited to this task as described above. In this case, the fine-tuned model with 50 training studies demonstrated a lesionwise sensitivity of 0.49 and lesionwise PPV of 0.59; such performance is likely insufficient for clinical utility at this stage of development, demonstrating that the choice of default model is very important. For reference, Krüger et al. reported a sensitivity of 0.60 and a lesionwise false-positive rate of 0.41 (PPV could not be calculated from the published data). While this glioma-specific model was chosen based on availability, a model pre-trained on pathology more similar to MS would likely demonstrate superior performance. In balancing sensitivity and specificity, in our practice, we envision that a high sensitivity model would be preferred, but this could easily vary based on the priorities and reading style of the interpreting radiologist.

## Data availability statement

The datasets presented in this study can be found in online repositories. The names of the repository/repositories and accession number(s) can be found at: https://github.com/wahligucsf/multiplesclerosis/blob/main/MS_Publish_Results.xlsx.

## Ethics statement

The studies involving human participants were reviewed and approved by University of California, San Francisco IRB (Laurel Heights Committee). Written informed consent from the participants’ legal guardian/next of kin was not required to participate in this study in accordance with the national legislation and the institutional requirements.

## Author contributions

SW, LS, and AR contributed to conception and design of the study. SW collected the training and testing cases. SW, AR, and JR manually segmented the cases. SW, PN, and DW implemented the deep learning segmentation algorithm. SW performed the statistical analysis. SW and AR wrote the manuscript. All authors contributed to the article and approved the submitted version.

## Footnotes

^1^ http://neuro.debian.net/pkgs/ants.html
