# Supplementary material for: 3D U-Net for automated detection of multiple sclerosis lesions: utility of transfer learning from other pathologies
Source: Front Neurosci. 2023 Oct 27;17:1188336. doi: 10.3389/fnins.2023.1188336 (PMC10641790; doi:10.3389/fnins.2023.1188336)
Supplement: Supplementary file 1 [file Image_1.pdf]

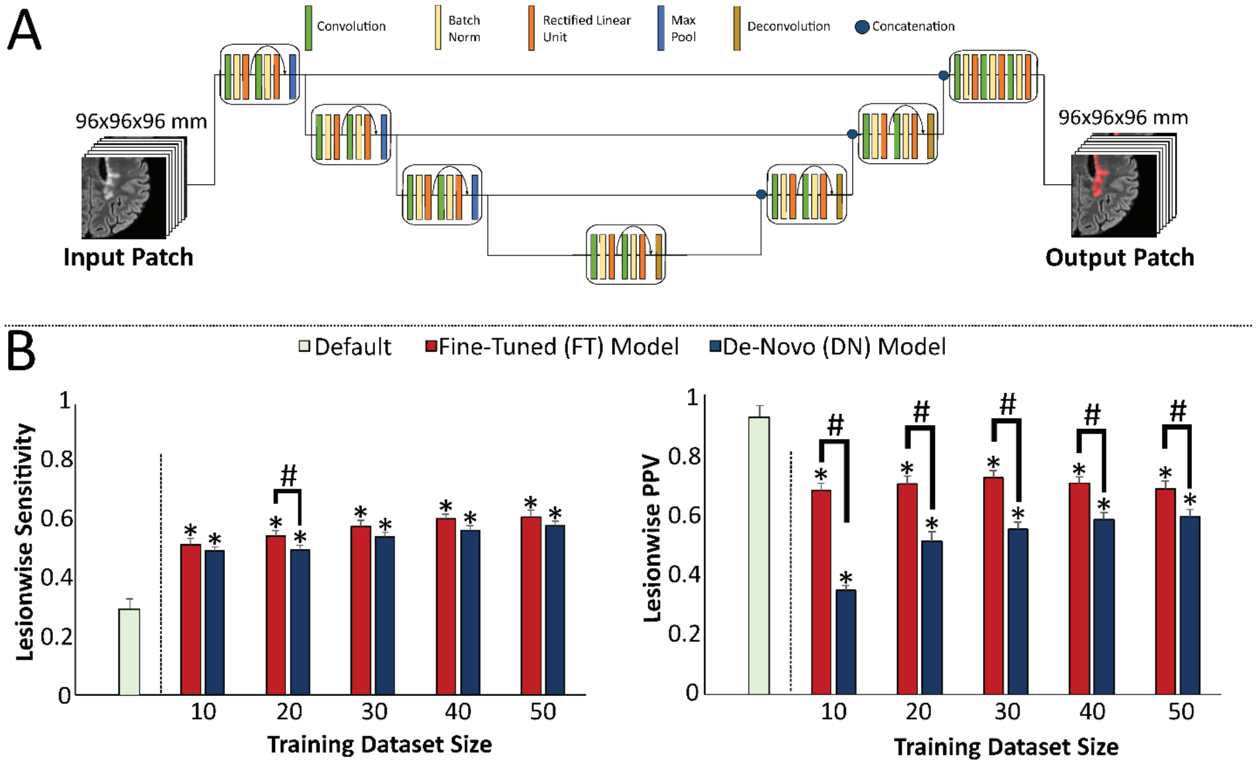

Supplementary Figure 1: Performance of single timepoint FLAIR lesion prediction models using an alternate shallow U-Net architecture. (A) Schematic of alternate shallow three-dimensional U-Net architecture. (B) Model performance assessed by lesionwise sensitivity and PPV statistics across the test set, compared to the default disease-invariant model (green). (Error bars in each bar graph represent +/- 1 standard error of the mean across patients. * = $p < 0.05$ in comparison with the default FLAIR model after multiple comparison correction. # = $p < 0.05$ in comparison with alternative training paradigm (fine-tune vs de-novo model) using the same training dataset size, after multiple comparison correction. FLAIR = fluid attenuation inversion recovery; PPV = positive predictive value.
