# Supplementary material for: 3D U-Net for automated detection of multiple sclerosis lesions: utility of transfer learning from other pathologies
Source: Front Neurosci. 2023 Oct 27;17:1188336. doi: 10.3389/fnins.2023.1188336 (PMC10641790; doi:10.3389/fnins.2023.1188336)
Supplement: Supplementary file 3 [file Image_3.pdf]

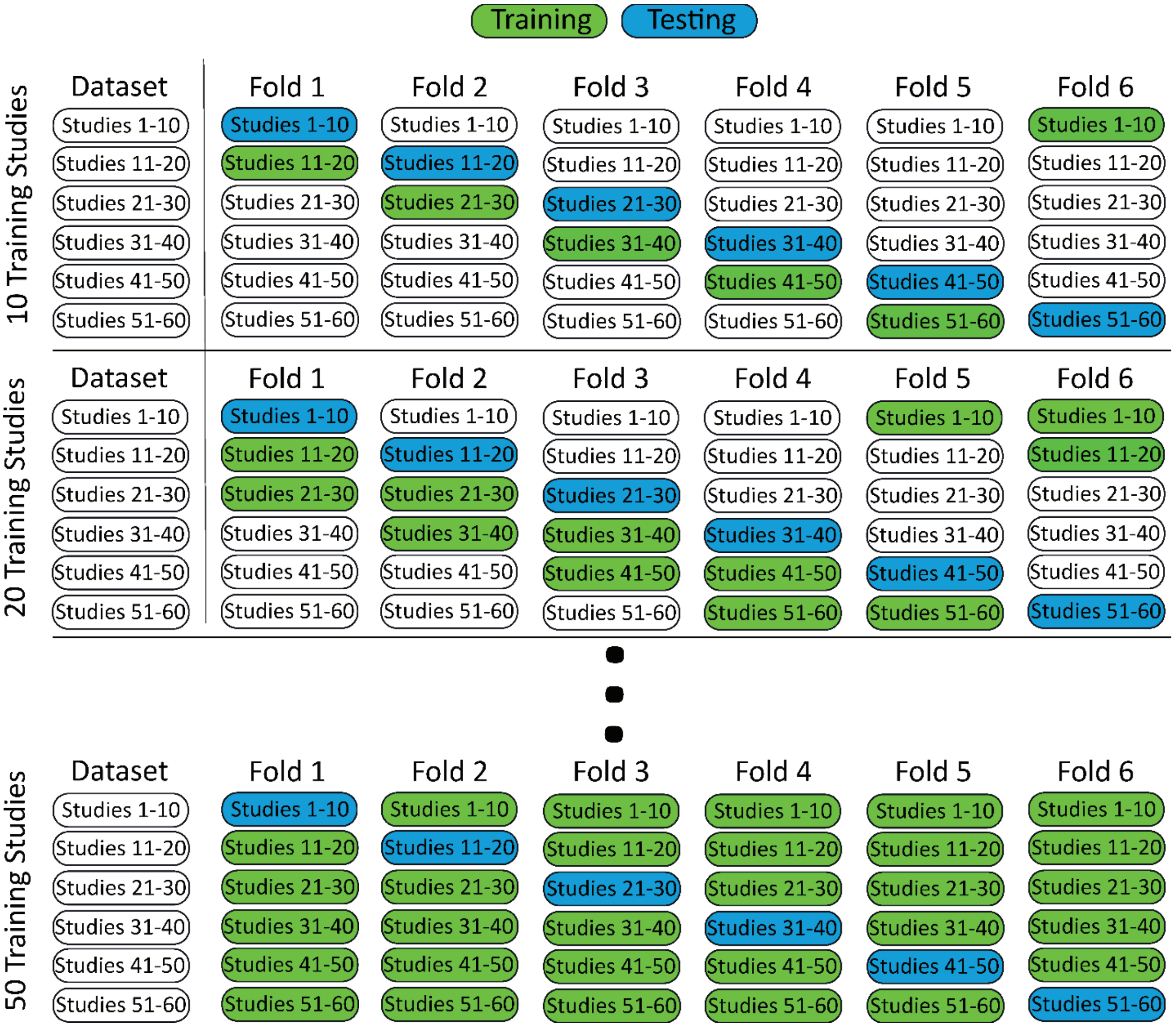


**Supplementary Figure 3:** Graphical depiction of 6-fold cross-validation approach. The 60 study dataset is divided into subsets of 10 studies. Each fold uses 1-5 subsets for training (labelled in green) and 1 subset for testing (labelled in blue).
