# Supplementary material for: 3D U-Net for automated detection of multiple sclerosis lesions: utility of transfer learning from other pathologies
Source: Front Neurosci. 2023 Oct 27;17:1188336. doi: 10.3389/fnins.2023.1188336 (PMC10641790; doi:10.3389/fnins.2023.1188336)
Supplement: Supplementary file 4 [file Table_1.pdf]

Model	Training Method	Lesionwise Sensitivity	$p^{Default}$	p^{Method}	Lesionwise PPV	$p^{Default}$	p^{Method}
**Default**		0.30±0.04			0.93±0.04		
**10**	FT	0.52±0.02	<0.001*	0.252	0.68±0.02	<0.001*	<0.001*
	DN	0.49±0.01	<0.001*		0.35±0.02	<0.001*	
**20**	FT	0.55±0.02	<0.001*	0.002*	0.70±0.03	<0.001*	<0.001*
	DN	0.50±0.02	<0.001*		0.51±0.03	<0.001*	
**30**	FT	0.58±0.02	<0.001*	0.028	0.72±0.02	<0.001*	<0.001*
	DN	0.54±0.02	<0.001*		0.55±0.02	<0.001*	
**40**	FT	0.61±0.02	<0.001*	0.017	0.70±0.02	<0.001*	<0.001*
	DN	0.57±0.02	<0.001*		0.58±0.02	<0.001*	
**50**	FT	0.61±0.02	<0.001*	0.059	0.68±0.03	<0.001*	<0.001*
	DN	0.58±0.01	<0.001*		0.59±0.02	<0.001*	

Supplementary Table 1: Single timepoint FLAIR segmentation model performance with alternate shallow U-Net architecture. All sensitivity and PPV values are displayed as mean ± standard error. FT = fine-tuned, DN = de-novo, PPV = positive predictive value, $p^{Default}$ = p value from comparison with the default model, p^{Method} = p value from comparison between fine-tuned and de-novo training methods with the same training dataset size. * indicates statistical significance ($p<0.05$ after accounting for Bonferroni correction).
